# Mediolateral episiotomy and risk of obstetric anal sphincter injuries and adverse neonatal outcomes during operative vaginal delivery in nulliparous women: a propensity-score analysis

**DOI:** 10.1186/s12884-022-04396-6

**Published:** 2022-01-19

**Authors:** Thomas Desplanches, Laetitia Marchand-Martin, Emilie-Denise Szczepanski, Marie Ruillier, Jonathan Cottenet, Denis Semama, Emmanuel Simon, Catherine Quantin, Paul Sagot

**Affiliations:** 1 Dijon University Hospital, Burgundy Perinatal Network, Pôle de Gynécologie-Obstétrique, Médecine Fœtale et Stérilité Conjugale, F-21000 Dijon, France; 2 Obstetrical, Perinatal and Pediatric Epidemiology Team (EPOPé), Center of Research in Epidemiology and Statistics (U1153), Team EPOPé U1153, Université de Paris, Inserm, Paris, France; 3 Dijon University Hospital, Service de Biostatistique et d’Informatique Médicale (DIM), F-21000 Dijon, France; 4 Inserm, CIC 1432, Clinical Epidemiology Unit, Dijon, France; 5 Dijon University Hospital, Clinical Investigation Center, Clinical Epidemiology Unit, Dijon, France; 6 Dijon University Hospital, Pôle de Pédiatrie, F-21000 Dijon, France; 7 Université Paris-Saclay, UVSQ, Univ. Paris-Sud, Inserm, High-Dimensional Biostatistics for Drug Safety and Genomics, CESP, Villejuif, France

**Keywords:** Operative vaginal deliveries, Episiotomy, Obstetric anal sphincter injuries, Adverse neonatal outcomes, Propensity score

## Abstract

**Background:**

The potential protective effect of mediolateral episiotomy for obstetrical anal sphincter injuries (OASIs) remains controversial during operative vaginal delivery because of the difficulties to take into account the risk factors and clinical conditions at delivery; in addition, little is known about the potential benefits of mediolateral episiotomy on neonatal outcomes.

The objectives were to investigate the associations between mediolateral episiotomy and both OASIs and neonatal outcomes, using propensity scores.

**Methods:**

We performed a retrospective population-based observational study from a perinatal registry that includes all births in a French region between 2010 and 2017. All nulliparous women with singleton pregnancy delivering by operative vaginal deliveries at 37 weeks gestational age or later were included. Inverse-probability-of-treatment weighting with propensity scores was used to minimize indication bias. OASIs was defined as third and fourth-degree tears according to Royal College of Obstetricians and Gynecologists. Two neonatal outcomes were studied: condition at birth (5-min Apgar score less than 7 and/or umbilical artery pH less than 7.10), and admission to neonatal intensive care unit.

**Results:**

The study population consisted of 7589 women; 2880 (38.0%) received mediolateral episiotomy. After applying propensity scores, episiotomy was associated with a lower rate of OASIs in forceps/spatula delivery (2.3 vs 6.8%, Risk Ratio (RR) 0.38, 95% Confidence Interval (CI) 0.28–0.52) and in vacuum delivery (1.3 vs 3.4%, RR 0.27, 95% CI 0.20–0.38) as compared with no episiotomy. Mediolateral episiotomy was associated with better condition at birth in case of forceps/spatula delivery (4.5 vs 8.8%, RR 0.56, 95% CI 0.39–0.81). In cases of fetal distress (40.7%), mediolateral episiotomy was associated with better condition of infant at birth in women who delivered by forceps/spatula (4.2 vs 13.5%, RR 0.52, 95% CI 0.31–0.89). No association was found with neonatal unit admission (RR 0.93, 95% CI 0.50–1.74).

**Conclusions:**

Use of mediolateral episiotomy was associated with a lower rate of OASIs during operative vaginal delivery, and in infants it was associated with better condition at birth following forceps/spatula delivery.

**Supplementary Information:**

The online version contains supplementary material available at 10.1186/s12884-022-04396-6.

## Background

Obstetric anal sphincter injuries (OASIs) are a rare but severe complication of vaginal delivery, with a prevalence between 0.25 and 6.0% [1]. These injuries have a major impact on women’s short-term and long-term health and well-being [2].

In 2016, 12.4% of deliveries in France were operative vaginal deliveries (OVD) [3], which is one of the most significant risk factors for OASIs [4]. OVD is more frequently performed in nulliparous than in multiparous women, and nulliparity is associated with the risk of OASIs [4]. The combination of these two risk factors contributes considerably to OASIs rates [5].

The potential protective effect of mediolateral episiotomy to prevent OASIs during OVD remains controversial, and several international guidelines recommend that mediolateral episiotomy “should be considered” [6–8]. In a pilot study for a planned randomized controlled trial that has addressed this issue [9], the authors concluded that a policy of routine episiotomy is not better or worse than a restrictive policy. However, the sample size was small, limiting the conclusions of this study. The results of observational studies are contradictory [10–20], and their conclusions may be limited owing to insufficient consideration of the confounding bias by indication. The implementation of a very restrictive episiotomy policy could even be associated with an increase in OASIs incidence during forceps delivery [21]. The major problem when evaluating OASIs is that maternal, fetal and medical characteristics are different in the episiotomy and no episiotomy groups. Previous studies have mostly used traditional covariate adjustment in regression models for risk adjustment. However, when there are great differences in important prognostic characteristics, adjusting for these differences with conventional multivariable techniques may not adequately balance the groups [22]. A propensity score (PSs) has been shown to effectively balance measured covariates between two groups in comparative observational studies [23].

In addition to the maternal complications associated with OVD, neonatal complications such as neonatal hypoxia may occur at birth [24]. Very few studies reported data on the association between episiotomy and adverse neonatal outcomes during OVD [9, 13, 14]. They found no association but the number of adverse neonatal outcomes was low, thus limiting the power of their analysis. The indication of episiotomy for fetal distress was regularly reported by obstetricians in several studies [25, 26]. It therefore seems relevant to study whether there is a difference in neonatal health status depending on whether or not a mediolateral episiotomy is performed.

Our objective was to assess the association between mediolateral episiotomy and both OASIs and adverse neonatal outcomes. The analysis in this study is focused on controlling for indication bias: we restricted our analysis to nulliparous women with singleton pregnancy delivering by OVD at term, and we used propensity scores to control for residual confounding by indication.

## Methods

### Study design and population study

This retrospective observational study was conducted in Burgundy, France, between January 2010 and December 2017. Over this 8-year period, 12 maternities managed approximately 17,000 births per year. Levels of care are based on a three-tiered system defined by national regulation. These facilities are gathered in the hierarchical Burgundy perinatal network (BPN), which was accredited by the regional health authorities in 2000.

All deliveries and terminations of pregnancies that occur within the BPN at or after 22 completed weeks of gestation and/or with a birth weight > 500 g have been systematically recorded in an anonymous database used to regularly assess the medical practices within the network. Maternal and neonatal medical data are prospectively recorded from the mandatory discharge abstracts for each hospitalized patient (used to determine the activity-based funding of French hospitals). Twenty additional specific perinatal characteristics, eleven for each mother and nine for each newborn, were also prospectively recorded. In accordance with European and French law, patient data have to be rendered anonymous in each maternity unit before being sent to the evaluation unit for data validation and mother/child linkage. The anonymization methods routinely used for BPN data were developed by our research team. ANONYMAT Software are also used in national applications [27].

In the current study, we restricted the analyses to nulliparous women who delivered a singleton live infant in cephalic presentation by an OVD at 37 weeks gestational age or later in 9 of the 12 maternity units in the region (five level-I maternity units, three level-II maternity units and one level-III maternity unit (university hospital)).

We excluded 3 hospitals from the analyses because data on parity, induction of labor, and Apgar score were not collected during the entire period in 2 hospitals, and the last hospital was closed during the study period.

### Outcomes and exposition measures

To classify OASIs, we used the Royal College of Obstetricians and Gynecologists classification [7], which is most widely used in the international literature. Only third-degree (defined as injuries of external and/or internal anal sphincter) and fourth-degree tears (defined as injuries of anal sphincter complex and anorectal mucosa) were taken into account and pooled for the analyses. OASIs was diagnosed by an obstetrician with a clinical examination (vaginal and rectal examination) of the perineum just after operative vaginal delivery. OASIs was identified in our database using the International Classification of Diseases 10th Revision (ICD-10 codes O702 - third degree perineal laceration during pregnancy and O703 - fourth-degree perineal laceration during pregnancy) and/ or the French Common Classification of Medical Procedures (CCAM codes JMCA001- Immediate suture of obstetrical tear of the perineum with lesion of the rectum, and JMCA003 - Immediate suture of obstetrical tear of the perineum with lesion of the external sphincter muscle of the anus). The first code is related to the diagnosis, and the second code corresponds to the surgical procedure.

Two adverse neonatal outcomes were studied: condition at birth, including low Apgar score (5-min Apgar score < 7) [28] and/or low arterial blood gases (umbilical artery pH < 7.10) [29, 30]; and admission to neonatal intensive care unit (NICU). Adverse neonatal outcomes were studied in the 2013–2017 period because umbilical artery pH values were systematically recorded from 2013 in the level II and III maternity units.

Mediolateral episiotomy was identified with the French Common Classification of Medical Procedures (code JMPA006).

### Statistical analysis

Maternal characteristics were compared in women who had episiotomy and women who did not, using Chi^2^ tests. The changes over time in the mediolateral episiotomy rate, the OVD rate and the OASIs rate were described using the Cochran-Armitage test.

To control confounding factors that might influence the use of episiotomy and the occurrence of OASIs, we used a PS approach. A woman’s PS was defined as the probability that she would have an episiotomy intervention based on her individual covariates [31]. The first step was to estimate a propensity score for all women using a logistic regression model with episiotomy as the dependent variable in relation to the following baseline maternal and obstetrical characteristics: induction of labor, prolonged pregnancy defined as more than 41^+ 0^ weeks of gestation, epidural analgesia, fetal distress during labor [32], occiput posterior position, mode of delivery (forceps and spatula delivery were grouped, and vacuum delivery), birth weight as a proxy of prenatal suspicion of large for gestational age, year of delivery, and level of maternity unit. Variables included in the PSs model were based on the literature [4].

Then, the inverse probability of treatment weighting (IPTW) based on estimated PSs was used to obtain a pseudo population in which treatment assignment is independent of measured baseline covariates [22]. The comparability of groups was verified by calculating standardized differences in the weighted samples. A standardized difference below 10% is considered an acceptable imbalance between groups [22].

We finally estimated the association between mediolateral episiotomy and OASIs using mixed log-poisson regression models, obtaining risk ratios (RRs) and 95% confidence intervals (CIs). We used multilevel modelling to take into account the hierarchical structure of the data (women within maternity units) and the non-independence of observations within maternity units.

We also investigated the associations between mediolateral episiotomy and adverse neonatal outcomes with similar methodologies. PSs were estimated using a logistic regression model that included all possible confounders likely to have affected neonatal outcomes, including maternal age, BMI, smoking, gestational diabetes, hypertension disorders in pregnancy (defined as gestational hypertension, and pre-eclampsia associated or not with complications such as HELLP syndrome, eclampsia and placental abruption), induction of labor, prolonged pregnancy, epidural analgesia, fetal distress, occiput posterior position, mode of delivery, small for gestational age as a proxy of prenatal suspicion of small for gestational age (SGA, defined as <10th percentile for gestational age) [33], year of delivery and level of maternity unit. We investigated the association of mediolateral episiotomy with two indicators of infant condition at birth: 5-min Apgar scores less than 7 and/ or umbilical artery pH less than 7.10, and admission to NICU.

Fetal distress during labor [32] is one the most frequently reported clinical indications for episiotomy use [25] and is known to be associated with infant condition at birth. We therefore performed stratified analyses according to fetal distress status. We recalculated PSs, and a second set of analyses compared the association between mediolateral episiotomy and condition at birth according to fetal distress status.

All of our analyses were stratified by the type of OVD (forceps/spatula or vacuum). Deliveries by spatula, an instrument for propulsion and direction, were grouped with forceps delivery [34].

Deliveries with sequential use of instruments were included in the forceps/spatula group, because they were not frequent and forceps are often used after a failed attempt at ventouse-assisted delivery*.*

Statistical significance was set with a two-tailed test at *p* < 0.05. All analyses were done with SAS v9.4 software.

### Missing data

Most of the variables used in this study had an exhaustiveness of 100% or less than 5% missing data, so the association between mediolateral episiotomy and OASIs was investigated on complete cases. However, the percentage of missing data for adverse neonatal outcomes was 9.4%. Analyses were first done on complete cases, and we then ran our models using multiple imputations (chained equations with a logistic regression imputation model for missing binary data and a multinomial imputation model for missing categorical data) [35]. Missing data were imputed by chained equations using the SAS “MI” procedure. Imputation model variables included maternal and neonatal characteristics: year of delivery, maternal age, smoking during pregnancy, body mass index, gestational diabetes, hypertension disorders in pregnancy, induction of labor, prolonged pregnancy, epidural analgesia, fetal distress, occiput posterior position, mode of delivery, birth weight, level of maternity unit, mediolateral episiotomy and outcomes. We generated 50 independent imputed datasets. A PSs was estimated for each of the generated datasets, and the results were pooled for a final analysis according to Rubin’s rules [35].

## Results

**Fig. 1:**
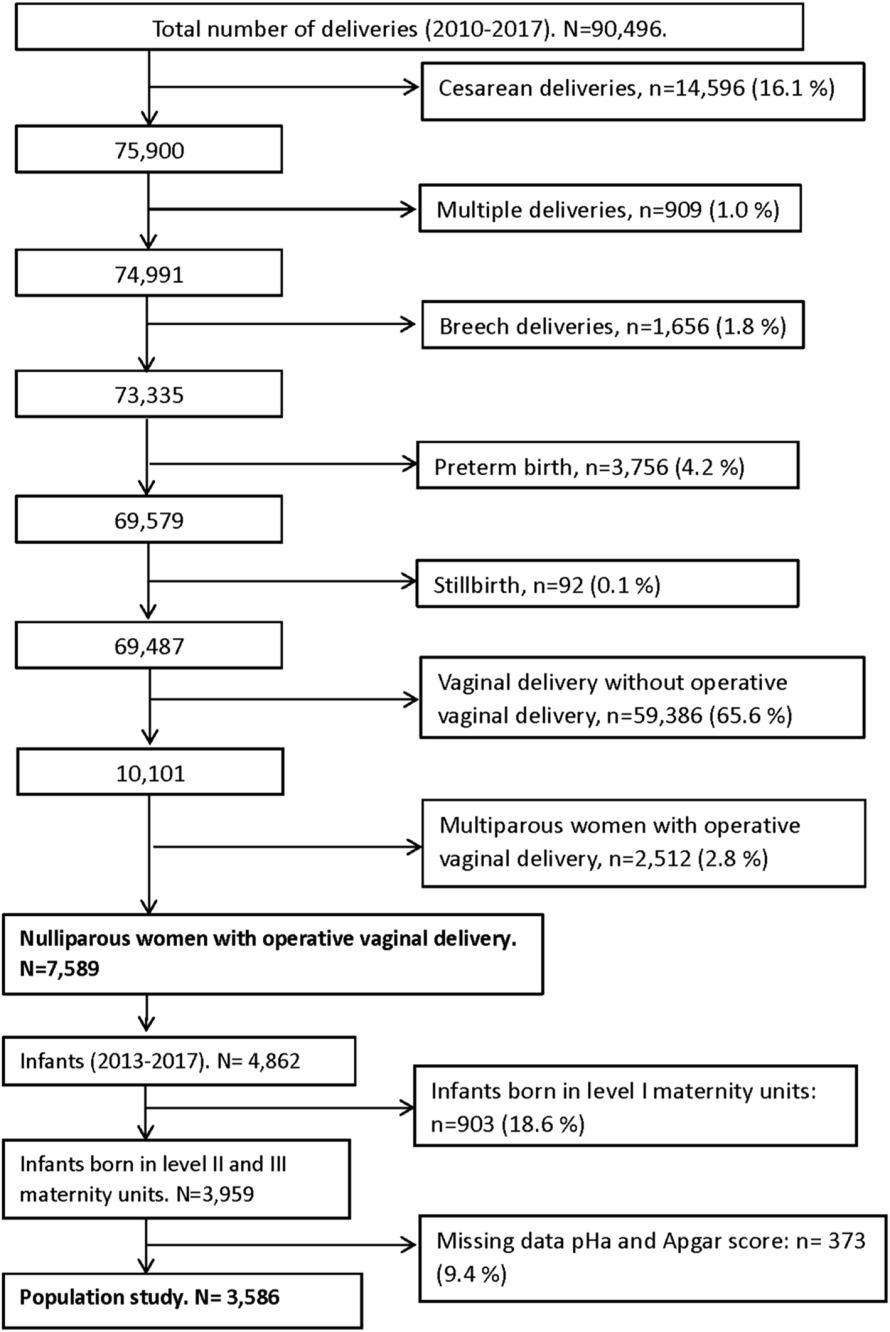
flow chart

We included 7589 nulliparous women who had an OVD of a single infant at term during the study period (Fig. 1). Vacuum delivery was more frequent than forceps/spatula delivery, respectively 64.3 and 33.7%. The proportion of vacuum delivery increased from 2010 to 2017 (Additional file: Table 1). The proportion of mediolateral episiotomy was 38.0% (2880). From 2010 to 2017, mediolateral episiotomy practices significantly decreased from 48.0 to 23.6% (Cochran-Armitage Test *P* < 0.001). The OASIs rate of 3.4% remained stable from 2010 to 2017 (Additional file: Table 1).

Maternal, labor and hospital characteristics for women with and without mediolateral episiotomy are presented in Table 1. Most of these characteristics differed significantly between the two groups. Episiotomy was more frequent in women requiring forceps/spatula extraction than vacuum extraction (53.1% (1433/2698) vs 29.3% (1421/4853), *P* < 0.01) (Table 1).

**Table 1:** Maternal, labor, neonatal and hospital characteristics without and with mediolateral episiotomy in nulliparous women with singleton pregnancy delivering by operative vaginal delivery at term

Characteristics, n (%)	Mediolateral episiotomy *N* = 2880	No mediolateral episiotomy *N* = 4709	*P* Value ^*^
Maternal characteristics			
Maternal age, years			0.08
< 25	933 (32.4)	1414 (30.0)	
25–34	1753 (60.9)	2954 (62.7)	
> 35	194 (6.7)	341 (7.3)	
Body mass index, kg/m^2^			0.01
< 18.5	183 (7.4)	314 (7.2)	
18.5–24.9	1597 (65.4)	2774 (63.7)	
25–29.9	456 (18.7)	812 (18.7)	
≥ 30	207 (8.5)	448 (10.3)	
Missing data	437	361	
Smoking during pregnancy	374 (13.0)	714 (15.1)	0.01
Gestational diabetes	225 (7.8)	478 (10.1)	0.01
Hypertension disorder of pregnancy	105 (3.6)	190 (4.0)	0.39
Labor, neonatal characteristics			
Induction of labor	581 (20.2)	1010 (21.5)	0.18
Gestational age at delivery > 41 WG	423 (14.7)	760 (16.1)	0.09
Epidural analgesia	2723 (94.6)	4241 (90.1)	0.01
Fetal distress during labor	1134 (39.4)	2034 (43.2)	0.01
Occiput posterior position	63 (2.1)	73 (1.6)	0.04
Operative vaginal delivery			0.01
Forceps/spatula delivery	1433 (50.2	1265 (26.9)	
Vacuum delivery	1421 (49.8)	3432 (73.1)	
Missing data	26	12	
Birth weight, g			0.01
< 2500	42 (1.5)	130 (2.8)	
2500–3999	2553 (91.9)	4362 (92.9)	
> 4000	184 (6.6)	199 (4.2)	
Missing data	1	18	
Small for gestational age < 10th percentile ^‡^	213 (7.4)	500 (10.6)	0.01
Hospital characteristics			0.01
Level I maternity unit	1093 (38.0)	693 (14.7)	
Level II maternity unit	966 (33.5)	2202 (46.8)	
Level III maternity unit	821 (28.5)	1814 (38.5)	

*WG* weeks of gestation. ^*^ The 2 groups were compared by the Chi^2^ tests. ^‡^ Growth curves adjusted for gestational age and gender. For each variable, percentages might not sum up to 100%, due to rounding

PSs were calculated and covariates were balanced in the two groups (standardized differences in the weighted samples were less than 10%).

Mediolateral episiotomy was associated with lower rates of OASIs in forceps/spatula delivery (2.3 vs 6.8%, Risk Ratio 0.38, 95% Confidence Interval (CI) 0.28–0.52) and in vacuum delivery (1.3 vs 3.4%, RR 0.27, 95% CI 0.20–0.38) (Table 2).

**Table 2:** Association between mediolateral episiotomy and OASIs according to the type of operative vaginal delivery

	Total number of women^†^	Mediolateral episiotomy		No Mediolateral episiotomy		RR (95% CI)	
		Number of women	Number (%) with OASIs	Number of women	Number (%) with OASIs	Univariate analysis *	Using IPTW ^*^
Whole population	7589	2880	52 (1.8)	4709	203 (4.3)	0.40 (0.29–0.56)	0.33 (0.27–0.41)
Forceps/spatula delivery	2698	1433	33 (2.3)	1265	86 (6.8)	0.34 (0.22–0.54)	0.38 (0.28–0.52)
Vacuum delivery	4853	1421	18 (1.3)	3432	117 (3.4)	0.31 (0.18–0.52)	0.27 (0.20–0.38)

*RR* risk ratio, *CIs* confidence intervals, *IPTW* inverse probability of treatment weighting, *OASIs* obstetric anal sphincter injuries. * Mixed model. ^†^ Missing data – mode of operative vaginal deliveries: *n* = 38. Covariates used to estimate the propensity score: induction of labour, epidural analgesia, occiput posterior position, prolonged pregnancy defined as > 41 weeks of gestation, fetal distress, type of instruments (forceps/spatula delivery and vacuum delivery), birth weight as a proxy of prenatal suspicion of large for gestational age, year of delivery and level of maternity unit

For neonatal outcomes, a total of 3586 infants were included (Fig. 1). Mediolateral episiotomy was associated with better condition at birth (pH umbilical artery < 7.10 or 5-min Apgar score < 7) in women who delivered by forceps/spatula (4.5 vs 8.8%, RR 0.56, 95% CI 0.39–0.81) (Table 3). This result was confirmed after multiple imputation (RR 0.63, 95% CI 0.40–0.99) (data not shown).

**Table 3:** Association between mediolateral episiotomy and the condition of infant at birth (pH umbilical artery < 7.10, 5-min Apgar score < 7) according to the type of operative vaginal delivery (2013–2017)

	Total number^†^	Mediolateral episiotomy		No Mediolateral episiotomy		RR (95% CI)	
		n	pH umbilical artery < 7.10 or 5-min Apgar score < 7, n (%)	n	pH umbilical artery < 7.10 or 5-min Apgar score < 7, n (%)	Univariate analysis ^*^	Using IPTW ^*^
Whole population	3586	966	55 (5.7)	2620	245 (9.4)	0.73 (0.54–1.00)	0.84 (0.70–1.01)
Forceps/spatula delivery	1141	702	20 (4.5)	439	62 (8.8)	0.56 (0.32–0.98)	0.56 (0.39–0.81)
Vacuum delivery	2442	526	35 (6.6)	1916	183 (9.6)	0.84 (0.57–1.23)	0.97 (0.79–1.20)

*RR* risk ratio, *CIs* confidence intervals, *IPTW* inverse probability of treatment weighting. ^*^ Mixed model after multiple imputation of missing data. ^†^ Missing data – mode of operative vaginal deliveries: *n* = 3. Covariates used to estimate the propensity score: maternal age, smoking, Body mass index, gestational diabetes, hypertension disorders in pregnancy, induction of labour, epidural analgesia, occiput posterior position, prolonged pregnancy, fetal distress, type of instruments (forceps/ spatula delivery and vacuum delivery), small for gestational age year of delivery, and level of maternity unit

In cases of fetal distress (40.7%), mediolateral episiotomy was again associated with better infant condition at birth in women who delivered by forceps/spatula (4.2 vs 13.5%, RR 0.52, 95% CI 0.31–0.89) (Table 4).

**Table 4:** Association between mediolateral episiotomy and the condition of infant at birth (pH umbilical artery < 7.10, 5-min Apgar score < 7) according to the type of operative vaginal delivery and fetal distress status (2013–2017)

	Total number^†^	Mediolateral episiotomy		No Mediolateral episiotomy		RR (95% CI)	
		n	pH umbilical artery < 7.10 or 5-min Apgar score < 7, n (%)	n	pH umbilical artery < 7.10 or 5-min Apgar score < 7, n (%)	Univariate analysis ^*^	Using IPTW ^*^
Forceps/spatula delivery							
Fetal distress	453	257	7 (4.2)	288	39 (13.5)	0.40 (0.16–0.98)	0.52 (0.31–0.89)
No fetal distress	688	274	13 (3.6)	414	23 (5.6)	0.80 (0.37–1.74)	0.70 (0.41–1.20)
Vacuum delivery							
Fetal distress	1008	228	21 (9.2)	780	112 (14.4)	0.83 (0.51–1.36)	0.89 (0.68–1.17)
No fetal distress	1434	298	14 (4.7)	1136	71 (6.3)	0.81 (0.45–1.47)	1.06 (0.77–1.48)

*RR* risk ratio, *CIs* confidence intervals, *IPTW* inverse probability of treatment weighting. ^*^ Mixed model after multiple imputation of missing data. ^†^ Missing data – mode of operative vaginal deliveries: *n* = 25. Covariates used to estimate the propensity score: maternal age, smoking, Body mass index, gestational diabetes, hypertension disorders in pregnancy, induction of labour, epidural analgesia, occiput posterior position, prolonged pregnancy, type of instruments (forceps/ spatula delivery and vacuum delivery), small for gestational age, year of delivery and level of maternity unit

We found no association between episiotomy and admission to NICU (Additional file: Table 2). Among the 83 infants admitted, 6 died.

## Discussion

Using a PS method, our study shows that whatever the type of OVD, mediolateral episiotomy was associated with a significantly lower rate of OASIs in nulliparous women with singleton pregnancy and OVD at term. Additionally, mediolateral episiotomy was associated with better infant condition at birth in case of forceps/spatula delivery, and in particular in cases of fetal distress.

In the present study, we used a rigorous IPTW method with PSs to minimize indication biases. The best current practices for the use of IPTW were followed [22]. The main confounding factors were included in our analysis. In addition, a mixed-model approach was used to take into consideration the clustering of births within hospitals. Our population was selected from a large regional registry which collected data prospectively and provided 8 years of reliable and recent data from level I, II and III maternity units belonging to the same perinatal network. The characteristics of our regional population, and the medical practices reported are similar to those observed in the France in 2016 [3].

This study has several limitations. First, this study was a retrospective observational study. Because randomization was impossible, we adjusted covariates available from the BPN database using IPWT methods. Second, additional unmeasured confounding factors might have affected the outcomes. We had no information regarding ethnicity as the collection of this information is not allowed in France. However, Asian women, a group at high risk of OASIs [4], make up a very small proportion of women who deliver in France. For instance, French census data suggests that only 1.7% of women who delivered in France in 2018 were born in Asia [36]. Data relative to the duration of second stage, operator experience and other interventions for perineal prevention such manual control of the expulsion and perineal support were not recorded. These practices are known to reduce the risk of perineal sphincter tears [37] and are almost systematically performed in France, as reported by a recent study [38]*.* Finally, we did not know how the episiotomy was actually performed although its technique is important [39]. To be protective, a mediolateral episiotomy should be at least 45° from the midline after suturing, which implies a minimum section of 60° [7]. The French guidelines have recommended this technique since 2005 [40]. However, it is difficult to record the accurate measurement of the incision angle because some discrepancies may exist between the self-reported section angle and the actual angle in current practice [41]. Third, the overall prevalence of OASIs in our study is close to the rate found in a previous cohort study [1]. However, it may be underestimated because ultrasound scans were not routinely used to collect cases of occult OASIs [42]*.* Fourth, the exclusion of 3 hospitals is likely to have a limited impact because the rates of OASIs and the main risk factors in these hospitals were not significantly different from those in our study. Our results can only be generalized to countries with a moderate restrictive policy of mediolateral episiotomy and to population with similar characteristics which represents a high-risk subgroup of OASIs. Finally, data on long-term complications are not available but they should be investigated to better inform women who require instrumental assistance to deliver.

Episiotomy rates for OVD vary across countries [1]. Our rate, which falls within the wide range of figures reported elsewhere, decreased during our study period (from 48 to 23%). This decrease was also observed in France as a whole [3], and it can be explained by the implementation of the French national obstetrical guidelines [40] and a decrease in the use of forceps/spatula in favor of vacuum, which is associated with lower episiotomy rates.

The protective effect of mediolateral episiotomy in women during OVD remains controversial: several observational studies have shown a lower rate of OASIs when an episiotomy was used, similar to our findings [10–12, 16], but other studies found no association [17, 19]. However, the authors of meta-analyses [16, 17] pointed out that the studies used for their analyses had some methodological limitations. To minimize potential indication biases, we used a PS method, similar to Ankarcrona et al. [20]. Compared with their results [20], we observed a greater reduction in the rates of OASIs in women who had an episiotomy compared with those who did not. The rate of episiotomy during vacuum delivery was similar to ours but their rate of OASIs was much higher (13.6%). No data was reported in forceps delivery.

Another important result of our study is that mediolateral episiotomy was associated with better infant condition after forceps/spatula delivery, and in particular in case of fetal distress. This result is particularly interesting considering that 5-min Apgar scores < 7 and/or umbilical artery pH < 7.10 are known to be associated with adverse neurological outcomes [28–30]. The proportion of fetal distress observed in our study was similar to previous studies [11, 13]. Fetal distress is recognized as one of the main indications for extraction [43] and may be related to these adverse neonatal outcomes. We hypothesize that the use of episiotomy in cases of fetal distress might reduce the duration of the second stage of labor, potentially improving the infant condition at birth after forceps/spatula delivery. Compared with the vacuum extractor, forceps use is more likely to result in a vaginal birth [43] and the duration tends to be slightly shorter [34, 44]. Forceps/spatula are used to guide the descending fetus by traction or propulsion, while a vacuum extractor is principally used for cephalic flexion, induction of rotation and comparatively less forceful traction [34]. Consequently, obstetricians can use these two instruments for different indications, which probably explains why we did not observe a significant association between episiotomy and neonatal outcomes in case of vacuum delivery.

Very few studies have investigated the association between episiotomy and neonatal outcomes in forceps deliveries [13, 14], and, while they found no association, the proportion of neonatal outcomes was low in these studies, thus limiting the power of their analysis. In addition, their episiotomy rates were much higher than in our study (90 and 53%, respectively). Consequently, additional studies are needed to confirm these results.

## Conclusion

Using recent prospective data and a propensity score to limit indication biases, we found that the use of mediolateral episiotomy was associated with a lower rate of OASIs in nulliparous women undergoing OVD. We also found that mediolateral episiotomy was associated with favorable infant condition at birth in case of forceps/spatula delivery, particularly in cases of fetal distress, which is one of the main indications for assisted vaginal birth.

Despite the concerns of women and the complications generated by the practice of episiotomy, mediolateral episiotomy may be a means to prevent OASIs during OVD and could improve the condition of the infant at birth after forceps/spatula delivery.

## Supplementary Information

**Additional file 1: Table S1.** Change over time in operative vaginal delivery, mediolateral episiotomy practices and OASIs in nulliparous women with operative vaginal delivery at term with live-born singletons. **Table S2.** Association between mediolateral episiotomy and admission to the neonatal intensive care unit according to the type of operative vaginal delivery (2013–2017).

## Data Availability

The datasets used and/or analyzed during the current study are available from the corresponding author on reasonable request.

## Abbreviations

BPN: Burgundy Perinatal Network
OASIs: Obstetric anal sphincter injuries
OVD: Operative vaginal deliveries
PSs: Propensity scores
