# Supplementary material for: Mediolateral episiotomy and risk of obstetric anal sphincter injuries and adverse neonatal outcomes during operative vaginal delivery in nulliparous women: a propensity-score analysis
Source: BMC Pregnancy Childbirth. 2022 Jan 19;22:48. doi: 10.1186/s12884-022-04396-6 (PMC8772124; doi:10.1186/s12884-022-04396-6)
Supplement: Supplementary file 1 — Additional file 1: Table S1. Change over time in operative vaginal delivery, mediolateral episiotomy practices and OASIs in nulliparous women with operative vaginal delivery at term with live-born singletons. Table S2. Association between mediolateral episiotomy and admission to the neonatal intensive care unit according to the type of operative vaginal delivery (2013–2017). [file 12884_2022_4396_MOESM1_ESM.docx]

Additional Table 1: Change over time in operative vaginal delivery, mediolateral episiotomy practices and OASIs in nulliparous women with operative vaginal delivery at term with live-born singletons.

|  | Total  (2010-2017) | 2010 | 2011 | 2012 | 2013 | 2014 | 2015 | 2016 | 2017 | *P* Value * | |
| --- | --- | --- | --- | --- | --- | --- | --- | --- | --- | --- | --- |
| Operative vaginal delivery, n (%) |  |  |  |  |  |  |  |  |  | 0.005 |  |
| Forceps/spatula delivery | 2,698 (33.7) | 321 (34.5) | 320 (35.5) | 391 (44.1) | 319 (38.1) | 297 (32.5) | 343 (34.7) | 400 (37.3) | 307 (30.0) |  |  |
| Vacuum delivery | 4,853 (64.3) | 609 (65.3) | 581 (64.4) | 496 (55.5) | 517 (60.3) | 616 (67.2) | 644 (65.1) | 672 (62.5) | 718 (70.0) |  |  |
| Mediolateral episiotomy, n (%) | 2,880 (38.0) | 448 (48.0) | 393 (43.6) | 402 (45.0) | 382 (44.6) | 364 (39.7) | 320 (32.4) | 329 (30.6) | 242 (23.6) | 0.001 |  |
| OASIs, n (%) | 255 (3.4) | 35 (3.4) | 30 (3.1) | 29 (3.4) | 25 (2.6) | 21 (2.1) | 30 (2.9) | 41 (3.5) | 44 (3.5) | 0.45 |  |

OASIs: obstetric anal sphincter injuries.

^*^ The change was compared by the Cochran-Armitage test.

Supporting Information for review and online publication only.

Additional Table 2: Association between mediolateral episiotomy and admission to the neonatal intensive care unit according to the type of operative vaginal delivery.

|  | Total number | Mediolateral episiotomy | | No Mediolateral episiotomy | | RR (95% CI) | |
| --- | --- | --- | --- | --- | --- | --- | --- |
|  |  | n | Admission to NICU,  n (%) | n | Admission to NICU,  n (%) | Univariate analysis ^*^ | Using IPTW ^*^ |
| Forceps/spatula delivery | 1,663 | 800 | 12 (1.5) | 863 | 13 (1.5) | 1.05 (0.51-2.20) | 0.92 (0.40-2.10) |
| Vacuum delivery | 3,162 | 812 | 15 (1.8) | 2,350 | 43 (1.8) | 1.12 (0.74-1.68) | 1.16 (0.83-1.62) |

RR: risk ratio, CIs: confidence intervals. IPTW: inverse probability of treatment weighting. NICU: neonatal intensive care unit. ^*^ Mixed model after multiple imputation of missing data.

Covariates used to estimate the propensity score: maternal age, smoking, body mass index, gestational diabetes, hypertension disorders in pregnancy, induction of labour, epidural analgesia, occiput posterior position, prolonged pregnancy, fetal distress, type of instruments, small for gestational age, year of delivery, and level of maternity unit.
